# mSphere of Influence: Discovering New Layers of Complexity in the Immune System

**DOI:** 10.1128/mSphere.00359-21

**Published:** 2021-05-05

**Authors:** Rebecca A. Drummond

**Affiliations:** a Institute of Immunology & Immunotherapy, Institute of Microbiology & Infection, University of Birmingham, Birmingham, United Kingdom

**Keywords:** central nervous system infections, fungi, macrophages, neuroimmunology, single-cell omics

## Abstract

Rebecca Drummond works in the field of antifungal immunity. In this mSphere of Influence article, she reflects on how papers by Amit et al. (H. Keren-Shaul, A. Spinrad, A. Weiner, O. Matcovitch-Natan, et al., Cell 169:1276–1290, 2017) and Ayres et al. (K. K. Sanchez, G. Y. Chen, A. M. P. Schieber, S. E. Redford, et al., Cell 175:146–158, 2018) made an impact on her by introducing her to new concepts in immune system complexity.

## Abstract (precis)

Rebecca Drummond works in the field of antifungal immunity. In this mSphere of Influence article, she reflects on how papers by Amit et al. (H. Keren-Shaul, A. Spinrad, A. Weiner, O. Matcovitch-Natan, et al., Cell **169**:1276–1290, 2017) and Ayres et al. (K. K. Sanchez, G. Y. Chen, A. M. P. Schieber, S. E. Redford, et al., Cell **175**:146–158, 2018) made an impact on her by introducing her to new concepts in immune system complexity.

## COMMENTARY

A textbook drawing of an immune response is relatively simple; an innate immune cell (such as macrophage) recognizes a pathogen via pattern recognition receptors (PRRs), kills it, and/or presents peptides from this invader to lymphocytes, leading to the activation of an adaptive immune response. In reality, the pathogen engages several PRRs on the macrophage, leading to the activation of multiple signaling pathways that cross-regulate each other, and these can be further influenced by a unique cocktail of metabolites and cytokines that are specific to the organ and type of infection. I am an infectious disease immunologist because I am drawn to this dizzying complexity of host-pathogen interactions. In recent years, new concepts of immune system complexity have been introduced to me via two publications. The findings within these papers have challenged my scientific thinking and are the source of inspiration for ongoing research within my laboratory, where we attempt to unravel host-pathogen interactions in the context of invasive fungal disease, a group of life-threatening infections that primarily affect vulnerable patients with underlying health disorders.

While working as a postdoctoral fellow, I read a publication by Amit and colleagues (1) that used single-cell RNA sequencing to examine responses of brain-resident macrophages (called microglia) within the Alzheimer’s brain. This approach identified a subset of microglia that associates with amyloid plaques, which they called “damage-associated microglia” (DAMs). Both mice and humans appear to generate DAMs in response to neuroinflammation, indicating that the presence of DAMs is an important biomarker for neurodegenerative diseases. This paper was my initial introduction to the world of single-cell-omics and the wealth of discovery that this technique may bring. The advent of single-cell RNA sequencing technology has led to several discoveries of novel immune cell subsets, leading to the identification of new disease biomarkers (2) and a greater understanding of how immune cells maintain homeostatic function of tissues (3). New technologies that build on this platform have also shown that mammalian cells can be defined by their lipid profiles (4), not just their transcriptomic signatures, opening up new concepts in how we define an immune cell functional phenotype.

I had become interested in microglial subsets while working on human CARD9 deficiency, a primary immunodeficiency disorder that causes the spontaneous development of fungal brain infections (5). We found that microglia required CARD9 to activate protective antifungal immune responses in the brain, and this helped to explain why other organs (which do not have microglia) were protected from fungal invasion in CARD9-deficient patients (6, 7). Reading about disease-specific states of microglia has driven me to question the heterogeneity of myeloid cell responses during fungal infection, and this is one of the main lines of inquiry in our laboratory. I am especially interested in this given that fungi often present a moving target for the immune system. Many fungal pathogens exist in a variety of morphological states, which each have a unique collection of PAMPs and antigens, resulting in divergent myeloid cell responses for different morphological forms (for example, only the filamentous forms of *Candida* and Aspergillus fungi activate inflammasomes [8, 9]). The use of single-cell-omics technologies presents an opportunity to define the heterogeneity of host immune cells and of fungal populations and how these might integrate to determine the outcome of infection. I am looking forward to future studies utilizing these technologies, since I believe they will provide a new dimension in our understanding of host-fungus interactions.

The second paper that has had a significant impact on my thinking was by Ayres and her team (10). This work asked a simple question: in a 50% lethal dose (LD_50_) challenge survival experiment, what was the difference between mice that survived the infection and the mice that died? Using a Citrobacter rodentium bacterial infection model, the authors showed that surviving mice had modulated iron metabolism, which led to increased glucose levels in the gut. This meant that the pathogenic bacteria did not need to compete with the host for glucose, which led to reduced expression of virulence factors. Using this information, the authors showed that dietary iron could be used to select for low-virulence strains of pathogenic bacteria and increase survival rates. This adaptation of the host to live with the infection, without clearing it, is termed tolerance, a term first coined by plant immunologists to describe infected plants that remain healthy to produce fruit. This paper presented a novel way to treat infections that did not rely on using antibiotics, where development of resistance can limit their long-term use. Therapeutic interventions that do not rely solely on antimicrobial drugs are especially important for fungal infections, where development of novel antifungal drugs is slow in part because of similar biochemistry between mammals and fungi, resulting in limited target availability (11). What inspired me most about this work was how an understanding of tolerance theory in plants led, in my opinion, to a significant shift in perspective for infectious disease immunology in mammals. We typically teach immunology students how the immune system recognizes and kills microbes to clear an infection, but this paper draws attention to different mechanisms that operate during infection that minimize damage caused by inflammation and/or microbial processes.

Collectively, these two papers have helped me think about my field in a wider context and inspired me to broaden my reading material into new fields. The concepts defined in these papers have contributed to the development of my independent research program, in which we hope to contribute new insights into the host-pathogen interactions occurring during invasive fungal diseases.
